# Case Report: successful treatment of severe bronchiectasis by inhalation of mesenchymal stem cell-derived exosomes

**DOI:** 10.3389/fmed.2025.1690559

**Published:** 2025-10-22

**Authors:** Yanyi Liu, Kun Xia, Haoxiang Zhang, Guangxi Li

**Affiliations:** Department of Respiratory, Guang'anmen Hospital, China Academy of Chinese Medical Sciences, Beijing, China

**Keywords:** bronchiectasis, inhalation, exosomes, mesenchymal stem cells, case report

## Abstract

Bronchiectasis is a chronic respiratory disorder characterized by irreversible airway dilation and recurrent infections, remains a therapeutic challenge. MSCs-exosomes based treatment is a potential treatment for respiratory disease; however, data regarding the efficacy of this novel therapy are currently lacking. We present a case of bronchiectasis treated with inhaled exosomes derived from human umbilical cord mesenchymal stem cells. As demonstrated in this patient, MSCs-exosomes inhalation may provide comprehensive benefits including reversal of hypoxemia, which could potentially result in enhancement of clinical outcomes and quality of life, and reduction in mortality.

## 1 Introduction

Bronchiectasis, a chronic disorder characterized by abnormal and permanent dilatation of the bronchi (1). Despite standard prolonged antibiotic therapy, approximately 50% of patients experience ≥2 annual exacerbations, with one-third requiring hospitalization (2). More severe and more frequent exacerbations accelerate lung function decline, impair quality of life, and mortality (3, 4), which poses a severe threat to human health.

Over 50% of bronchiectasis patients develop airflow obstruction with daily dyspnea, which is a strong mortality predictor (5). While bronchodilators are commonly used, evidence supporting their efficacy remains limited. A randomized trail reported that (6) 6-month tiotropium therapy failed to improve exacerbation frequency, symptoms, quality of life, or inflammatory markers. The treatment of such patients remains challenging, especially in severe cases.

The pathogenesis of bronchiectasis is complex, potentially underlying host defense defects, chronic bronchial infection, inflammation, mucociliary dysfunction and structural lung damage (1, 7). Pathogens evade immune clearance, perpetuating a destructive cycle of infection and inflammation. This complexity underscores the need for novel therapies. Here, we report a case detailing great improvements in bronchiectasis following inhaled human umbilical cord-derived mesenchymal stem cell exosomes (HucMSC-Exos) therapy.

## 2 Case presentation

A 53-year-old male with a 30-year smoking history (quit in 2015) presented in February 2022 with persistent productive cough and daily expectoration of approximately 40 mL purulent sputum, occasionally containing blood streaks or frank hemoptysis. High-resolution computed tomography (HRCT) demonstrated bilateral bronchiectasis with superimposed infection. Notably, the scan also revealed patchy consolidations and cavities, highly suggestive of pulmonary tuberculosis sequelae. Although the patient declined further testing, the bronchiectasis was deemed likely secondary to previous tuberculosis infection. Intermittent antibiotic therapy provided only symptomatic relief without achieving disease control. The patient subsequently developed progressive respiratory deterioration, manifested by declining oxygenation (peripheral capillary oxygen saturation (SpO₂) 90% on room air), severe pulmonary function impairment (VC max 1.63 L, FEV1 0.73 L, and FEV1/VC max 44.56%), and debilitating clinical symptoms including severe dyspnea (ambulation limited to ≤100 m by breathlessness), and significant weight loss [Body mass index (BMI): 14.71]. Consequently, ambulatory oxygen therapy was initiated in April 2023, followed by adjunctive traditional Chinese medicine in July 2023. Due to suboptimal adherence, the patient only received azithromycin intermittently.

Given the lack of clinical improvement, the patient initiated nebulized inhalation therapy with HucMSC-Exos (Harbin Huafang Biotechnology Co., Ltd., China) on March 28, 2024. Human umbilical cord mesenchymal stem cells (HucMSC) were obtained from a commercial cell bank and cultured under optimal conditions. Exosomes were isolated using ultracentrifugation and molecular sieve-based differential centrifugation. The extracted HucMSC-Exos were resuspended in 5 mL 0.9% saline for clinical application, with a dosage range of 1 × 10^10^–1 × 10^11^ particles per administration. The procurement, isolation, and culture of HucMSCs were approved by the institutional ethics committee. Treatment was administered twice weekly (3-day intervals) over 4-week courses. By May 18, 2024, respiratory function had improved significantly, with SpO₂ reaching 95% on room air, prompting initiation of the second treatment course. A third course was subsequently administered beginning June 13, 2024.

**Figure 1:**
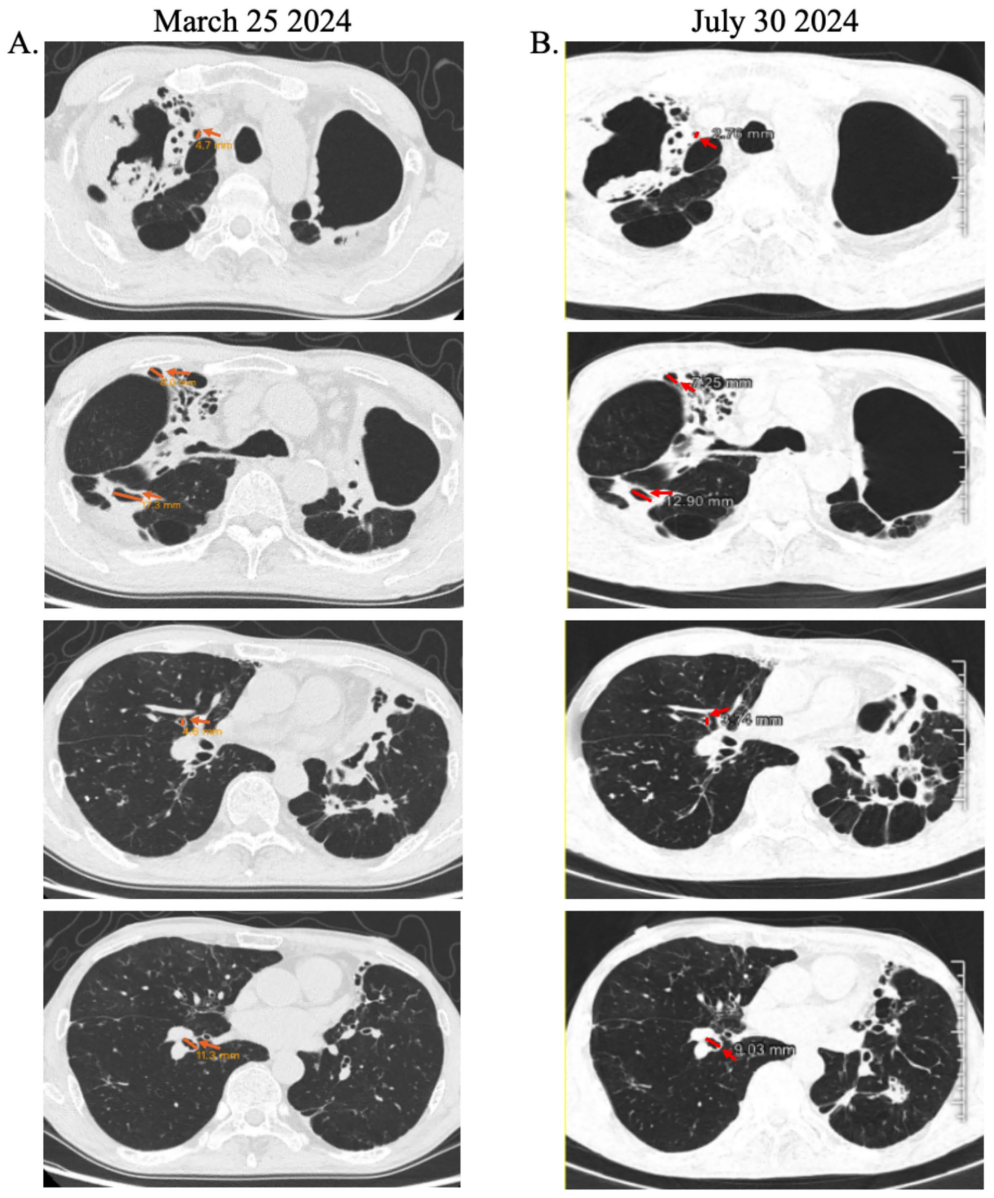
Radiographic evidence of air cavity reduction following HucMSC-Exos inhalation therapy. **(A)** Baseline chest CT scan demonstrating extensive cavitary lesions (yellow arrows), **(B)** follow-up scan showing significant reduction in cavity dimensions (red arrows).

Following three courses of HucMSC-Exos nebulization, the patient was successfully weaned off supplemental oxygen. By August 1, 2024, he exhibited marked clinical improvement in cough, sputum production, dyspnea, activity tolerance (3–5 miles daily), and appetite (BMI: 15.22). His SpO₂ stabilized at 97% on room air. Chest CT revealed partial reduction in cavity size compared to baseline (Figure 1). Post-treatment hematologic analysis showed decreased WBC, neutrophil, monocyte counts, and C-reactive protein, while renal function remained normal, supporting the safety of exosome inhalation (Table 1). Given sustained clinical stability, exosome therapy was discontinued with planned follow-up.

**Table 1:** Clinical laboratory changes of the patient before and after HucMSC-Exos inhalation.

Laboratory value	March 25 2024	July 30 2024
WBC count, × 10^9^/L	10.16	9.0
Neutrophil count, × 10^9^/L	7.18	6.3
Lymphocyte count, × 10^9^/L	1.8	1.8
Monocyte count, × 10^9^/L	0.75	0.6
C-reactive protein, mg/L	24.28	9.0
Alanine aminotransferase (U/L)	5.7	9
Aspartate aminotransferase (U/L)	12.0	17
Alkaline phosphatase (U/L)	79	82
γ-gamma-glutamyl transferase (U/L)	15.7	19
Total bilirubin (μmol/L)	11.8	12.6
Albumin (g/L)	39.8	45.5
Creatinine (μmol/L)	59	55
Uric acid (μmol/L)	363	391
Urea (mmol/L)	5.4	5.3

At the December 2024 evaluation, the patient reported only occasional productive cough without hemoptysis or acute exacerbations. He maintained full work capacity and unrestricted travel ability. SpO₂ persisted within 96–98% on room air, and body weight increased by 7 kg versus pre-treatment (BMI: 17.10). Pulmonary function tests also demonstrated modest but consistent improvements (VC max 1.84 L, FEV1 0.97 L, and FEV1/VC max 54%) (Table 2).

**Table 2:** Lung function trends in the patient before and after HucMSC-Exos inhalation.

Lung function	April 19 2023	December 16 2024	June 26 2025
VC MAX (L)	1.63 (42.3%)	1.84 (43.0%)	1.89 (48.0%)
FVC (L)	1.63 (44.3%)	1.81 (44.0%)	1.61 (41.0%)
FEV 1 (L)	0.73 (23.4%)	0.97 (29.0%)	0.90 (27.0%)
FEV 1% FVC (%)	44.56%	54.00%	56.00%
FEV 1% VC MAX	44.56%	53.59%	47.61%
MEF 75 (L/s)	0.61 (7.9%)	1.44 (19.0%)	1.37 (18.0%)
MEF 50 (L/s)	0.32 (7.1%)	0.44 (10.0%)	0.39 (9.0%)
MEF 25 (L/s)	0.19 (11.5%)	0.21 (13.0%)	0.23 (14.0%)
MEF 25–75 (L/s)	0.29 (8.9%)	0.40 (11.0%)	0.40 (12%)
DLCO SB (mmol/min/kPa)	Failed to measure	4.15 (44%)	5.63 (57%)

By June 2025, the patient maintained an SpO₂ of 96%, and chest CT revealed no signs of infection. Notably, pulmonary diffusion capacity also showed significant improvement. (DLCO SB 57%) (Table 2).

## 3 Discussion

This case demonstrates significant clinical improvement in severe bronchiectasis with hypoxemia following HucMSC-Exos inhalation therapy. Meanwhile, the patient fully adhered to the prescribed treatment regimen and no adverse events were recorded during the entire treatment and follow-up period, underscoring the safety and tolerability of this novel approach.

Dyspnea in bronchiectasis arises from multifactorial mechanisms including airflow obstruction, impaired gas transfer, exercise deconditioning and the impact of comorbidities (1). Importantly, dyspnea severity independently predicts exacerbation frequency, hospital admissions, quality of life, and mortality (8, 9). While bronchodilators are mainstays in asthma and chronic obstructive pulmonary disease management, their efficacy in bronchiectasis remains controversial, with limited and indirect evidence supporting long-term use (10).

Mesenchymal stem cells (MSCs) represent a promising therapeutic approach for lung inflammatory diseases (11, 12). As key mediators of MSC paracrine effects, exosomes offer advantages including rapid self-renewal, enhanced proliferation capacity, and reduced immunogenicity compared to whole-cell therapies (13). Their cargo of bioactive molecules facilitates intercellular communication and host cell modulation (14), making them particularly attractive for bronchiectasis treatment.

The therapeutic potential of HucMSC-Exos in bronchiectasis remains underexplored. HucMSC-Exos may modulate disease progression through dual mechanisms of inflammation inhibition and structural repair promotion. The potent anti-inflammatory effects of HucMSC-Exos, documented through reduced inflammatory cell infiltration, decreased pro-inflammatory cytokine levels, and macrophage polarization modulation in acute lung injury (ALI), asthma and bronchopulmonary dysplasia (BPD) (15–17), may be underpinned by specific miRNA-mediated mechanisms. A key example is HucMSC-derived exosomal miR-451, which can alleviate inflammation by promoting macrophage M2 polarization via targeting the MIF-PI3K-AKT pathway (17), a process that could have contributed to the observed resolution of inflammation in our patient. Furthermore, HucMSC-Exos demonstrate structural and functional lung protective effects, including reduced airway resistance and increased FEV1 in silicosis models (18), decreased radiographic lung density in BPD, and attenuated apoptosis of alveolar epithelial and endothelial cells in emphysema (19). The molecular basis for this reparative capacity is becoming clearer. Studies in emphysema models show that (19) specific miRNAs carried by HucMSC-Exos, have been identified as key mediators in counteracting emphysematous changes and cell apoptosis. This evidence provides a compelling mechanistic rationale for how HucMSC-Exos inhalation might have contributed to the enhancement of lung ventilation and tissue integrity in our case, a condition also characterized by structural airway damage.

Nebulized drug delivery represents an optimal therapeutic strategy for pulmonary diseases, offering targeted lung deposition with maximal local bioavailability and minimal systemic exposure. This non-invasive approach is particularly advantageous for exosome therapy, as demonstrated by comparative studies showing inhaled exosomes achieve comparable efficacy to pirfenidone in pulmonary fibrosis models, with superior sustained-release properties (20). Our clinical observations further support the therapeutic potential of nebulized exosome delivery, as evidenced by the significant improvement of this patient.

## 4 Conclusion

To our knowledge, this represents the first reported case of clinically significant improvement in bronchiectasis following HucMSC-Exos inhalation therapy. While no randomized clinical trial data currently exist regarding HucMSC-Exos for bronchiectasis, our observed therapeutic outcomes suggest potential for disease modification. Prospective studies are warranted to evaluate the long-term efficacy and safety of this novel therapeutic approach.

## Data Availability

The original contributions presented in the study are included in the article/supplementary material, further inquiries can be directed to the corresponding author.
